# Association of hyperglycemia ratio and ventricular arrhythmia in critically ill patients admitted to the intensive care unit

**DOI:** 10.1186/s12872-023-03208-9

**Published:** 2023-04-28

**Authors:** Hechen Shen, Song Wang, Chong Zhang, Wenqing Gao, Xiaoqiong Cui, Qiang Zhang, Yuheng Lang, Meng Ning, Tong Li

**Affiliations:** 1 The Third Central Clinical College of Tianjin Medical University, Tianjin, China; 2 Department of Heart Center, The Third Central Hospital of Tianjin, Tianjin, China; 3 Tianjin Key Laboratory of Extracorporeal Life Support for Critical Diseases, Tianjin, China; 4 Tianjin ECMO Treatment and Training Base, Tianjin, China; 5 Artificial Cell Engineering Technology Research Center, Tianjin, China; 6 School of Medicine, Nankai University, Tianjin, China; 7 Nankai University Affiliated Third Center Hospital, Nankai University, Tianjin, China

**Keywords:** Stress hyperglycemia, Stress hyperglycemia ratio, Ventricular arrhythmia, Critically ill patient

## Abstract

**Introduction:**

The relationship between relative hyperglycemia and ventricular arrhythmia (VA) in critically ill patients admitted to intensive care units (ICU) remains unclear. This study aims to investigate the association between stress hyperglycemia ratio (SHR) and VA in this population.

**Methods:**

This retrospective and observational study analyzed data from 4324 critically ill patients admitted to the ICU, obtained from the Medical Information Mart for Intensive Care IV (MIMIC-IV) database. The SHR was calculated as the highest blood glucose level during the first 24 h of ICU admission divided by the admission blood glucose level. Based on the optimal cut-off values under the receiver operating characteristic curve, patients were stratified into high SHR (≥ 1.31) and low SHR (< 1.31) group. To investigate the impact of diabetes mellitus (DM) on the outcome, patients were stratified as low SHR/DM; low SHR/non-DM; high SHR/DM, and high SHR/non-DM. Restricted cubic spline (RCS) and logistic regression analysis were performed to analyze the relationship between SHR and VA.

**Results:**

A total of 4,324 critically ill patients were included in this retrospective and observational study. The incidence of VA was higher in the high SHR group. Multiple-adjusted RCS revealed a “J-shaped” correlation between SHR and VA morbidity. The logistic regression model demonstrated that high SHR was associated with VA. The high SHR/non-DM group had a higher risk of VA than other groups stratified based on SHR and DM. Subgroup analysis showed that high SHR was associated with an increased risk of VA in patients with coronary artery disease.

**Conclusion:**

High SHR is an independent risk factor and has potential as a biomarker of higher VT/VF risk in ICU-admitted patients.

**Supplementary Information:**

The online version contains supplementary material available at 10.1186/s12872-023-03208-9.

## Introduction

Ventricular arrhythmia (VA) is a significant clinical adverse event that is prevalent in patients admitted to the intensive care unit (ICU) and can be a direct cause of sudden cardiac death in hospitals. Timely defibrillation treatment is critical in restoring normal cardiac rhythms in cases of critical ventricular arrhythmias. Therefore, it is imperative to explore relevant biomarkers for predicting and preventing VA.

Stress hyperglycemia (SH) is a physiological reaction that occurs in patients with severe diseases without diabetes mellitus (DM) due to stress [1]. Higher SH has been associated with a poor prognosis [2, 3]. SH was previously diagnosed based on blood glucose concentration at admission [2]. However, elevated blood glucose levels in patients with DM admitted to the ICU can be caused by either the physiological reaction to severe disease, chronic poor glycemic control, or both, which are clinically indistinguishable [4, 5]. Consequently, Roberts et al. proposed a novel definition of SH using the stress hyperglycemia ratio (SHR) [6]. SHR is calculated based on the absolute levels of blood glucose and glycosylated hemoglobin (HbA1c) [6]. HbA1c reflects the prior glucose status over the past three months, which is not easily affected by acute illness. Therefore, accurate quantification of SH using SHR is a promising approach.

Apart from underlying ischemic or structural heart diseases, there is evidence suggesting that glucose levels play a crucial role in the diagnosis of VA, particularly in ICU patients [7, 8]. Extremely high or low glucose levels have been found to lead to QT interval prolongation and increased QT dispersion, reflecting abnormal ventricular myocardial repolarization [9]. To date, no study has explored the relationship between SHR and VA. The objective of this study was to investigate the association between SHR and VA in critically ill patients admitted to the ICU.

## Materials and methods

### Study population

This retrospective and observational study included a total of 4,324 critically ill patients. Data on initial glucose and glycated hemoglobin values were obtained from the Medical Information Mart for Intensive Care IV (MIMIC-IV) database at PhysioNet, within 24 h of their ICU admission. MIMIC IV is publicly available, longitudinal, and extensive critical care database approved by the ethics committee of Beth Israel Deaconess Medical Center and MIT. The first Author (HS) was certified (Record ID: 49,784,899) to access the database. Since all identifiable personal information was removed, patient-informed consent was not required. Information on the first hospitalization was used for patients frequently admitted to the ICU. Exclusion criteria were as follows: (1) Patients ≤ 18 years old (n = 30); (2) Patients lacking either glucose or Hb1Ac data on their first day in the ICU (n = 16,947); (3) Patients who were discharged or died within 24 h (n = 1,263).

### Variable extraction

Demographic information, vital signs, laboratory indices, and comorbidities data for patients’ first day of admission to the ICU were extracted from the MIMIC-IV database using PostgreSQL (version 14.5) and Navicat Premium (version 15.0) software. The extracted data included: (1) demographic information: age, gender, race, body mass index (BMI), and ICU type; (2) vital signs: respiration, heart rate, systolic blood pressure, diastolic blood pressure, and blood oxygen saturation (SpO2); (3) laboratory indices: hemoglobin, white blood cell, platelet, glucose, HbA1c, lactic acid, creatinine, potassium, and sodium levels; (4) Comorbidities: coronary artery disease(CAD), DM, peripheral vascular disease, hypertension, chronic obstructive pulmonary disease (COPD), and congestive heart failure. ICD-9 and ICD-10 codes were used together to determine the diagnosis of comorbidities.

### SHR and VA

To calculate the average chronic glucose levels, we used the following formula: [(28.7*HbA1c%) − 46.7] /18.8 [10]. To calculate SHR, we divided the fasting blood glucose on the first day of ICU admission by the estimated mean chronic blood glucose and expressed the result as a percentage. The diagnosis of VA including ventricular tachycardia (VT) (both non-sustained and sustained VT) and ventricular fibrillation (VF), was made based on the heart rhythm recorded by the electrocardiograph monitor during the patient’s ICU hospitalization.

### Statistical analysis

The optimal cut-off value of SHR for VA was determined using the receiver operating characteristic curve (ROC). T-tests, Pearson’s chi-square, and Fisher’s exact test were used for intergroup comparison. Baseline characteristics were presented as average (standard deviation) or median (interquartile range) for continuous variables and quantities (percentage) for categorical variables. Restricted cubic spline (RCS) was created to explore the dose-response association between SHR and VA. Subsequently, SHR was stratified according to DM and SHR cut-off value as follows: low SHR/DM; low SHR/non-DM; high SHR/DM; high SHR/non-DM). A multivariate logistic hazards model was created to analyze the relationship between VA and SHR among these groups. Subgroup analysis was conducted to verify the effect of SHR on VA in subsets of participants based on gender, age (< 60 and ≥ 60 years), BMI (< 30 and ≥ 30 kg/m^2^), CAD, hypertension, and DM. All analyses were performed using Stata SE.15.0 and GraphPad Prism v8.0. A two-tailed *P*-value of < 0.05 was considered statistically significant.

## Results

**Fig. 1:**
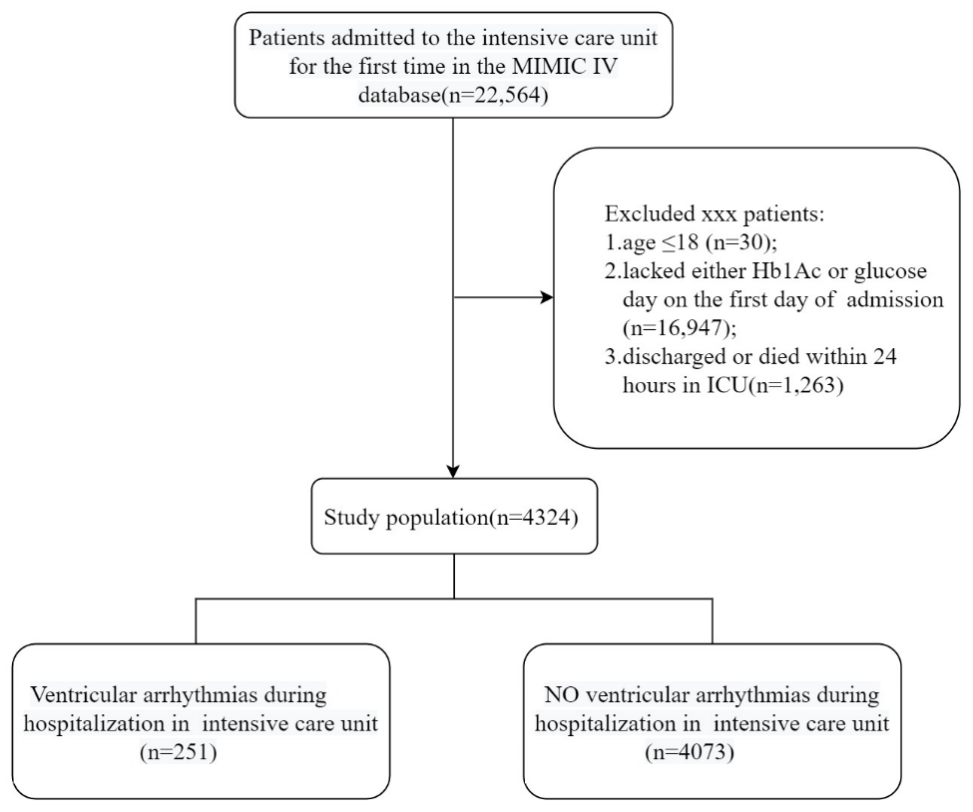
Flow chart of the screening of patient selection

The study initially obtained data from 22,564 hospitalized patients in the MIMIC IV database, but after applying the exclusion criteria, a final cohort of 4,324 eligible patients was selected for analysis. Figure 1 depicts the flow chart of the patient selection. An ROC curve analysis was conducted to determine the optimal cut-off value of SHR for VA. Using a cut-off value of 1.31, with a sensitivity of 41%, specificity of 75%, and Youden index of 0.161, the study population was stratified into low SHR (< 1.31) and high SHR (≥ 1.31) groups.

### Baseline characteristics

The mean age of the enrolled patients were 66.93 ± 13.22 years, of which, 2,852 (65.9%) were males. The mean SHR index on the first day of ICU admission was 1.15 ± 0.36. The baseline characteristics of patients with and without VA are shown in **Supplemental Table 1**. Patients in the VA group were more likely to be male, diagnosed with CAD and congestive heart failure, and admitted to the CCU compared with those in non-VA group. Additionally, patients in the VA group had lower heart rates and systolic blood pressure and showed higher levels of glucose, creatinine, and SHR within 24 h.

Moreover, 251 patients (5.8%) developed VA, with 129 in the low SHR group and 122 in the high SHR group. The incidence of VA was nearly 2-fold higher in the high SHR group compared to the low SHR group (Table 1). VT and VF-related morbidity were also investigated, and found to be significantly different between the two groups. Additionally, patients in the high SHR group had worse outcomes, including longer lengths of stay and a higher incidence of death in the ICU.

**Table 1:** Baseline characteristics and outcomes of patients based on the stress hyperglycemia ratio

Variables	Low SHR (n = 2857)		High SHR(n = 1467)	*P* value
Demographics				
Age (years)	67.6 ± 12.6		65.6 ± 14.2	<0.001
Gender, male, n (%)	1921 (67.2%)		931 (63.5%)	0.013
Race, n (%)				0.003
White	1890(66.2%)		893(60.9%)	
Hispanics	16(0.6%)		5(0.3%)	
Black	153(5.4%)		99(6.7%)	
Others	798(27.9%)		470(32.0%)	
Body mass index (kg/m^2^)	29.1 ± 6.2		29.2 ± 6.7	0.67
First Care Unit, n (%)				< 0.001
CVICU	2141 (74.9%)		581 (39.6%)	
CCU	222 (7.8%)		285 (19.4%)	
MICU/SICU	252 (8.8%)		373 (25.4%)	
NSICU	177 (6.2%)		113 (7.7%)	
TSICU	65 (2.3%)		115 (7.8%)	
**Vital signs**				
Respiratory Rate(beats/minute)	17.9 (16.2, 19.8)		18.8 (16.8, 21.4)	< 0.001
Heart rate (beats/minute)	80.1 (73.7, 87.5)		83.0 (74.4, 93.2)	< 0.001
Systolic blood pressure(mmHg)	113.4 (106.9, 121.2)		114.3 (106.0, 126.1)	0.083
Diastolic blood pressure(mmHg)	58.7 (53.5, 64.8)		60.8 (54.3, 68.6)	< 0.001
SpO2 (%)	97.7 (96.5, 98.7)		97.7 (96.2, 98.8)	0.40
**Comorbidities**				
CAD, n (%)	1868 (65.4%)		694 (47.3%)	< 0.001
COPD, n (%)	139 (4.9%)		89 (6.1%)	0.094
DM, n (%)	895 (31.3%)		473 (32.2%)	0.54
Hypertension, n (%)	930 (32.6%)		363 (24.7%)	< 0.001
Congestive heart failure, n (%)	880 (30.8%)		553 (37.7%)	< 0.001
Peripheral vascular disease, n (%)	412 (14.4%)		238 (16.2%)	0.12
**Severity of illness score**				
SOFA score	5(3,7)		6(4,9)	< 0.001
OASIS score	31(26,37)		34(28,41)	< 0.001
SIRS score	3(2,3)		3(2,3)	< 0.001
**Laboratory tests**				
Hemoglobin (g/dL)	11.5 (10.3, 12.8)		11.9 (10.4, 13.5)	< 0.001
White blood cell (K/µL)	14.5 (10.7, 18.4)		14.9 (11.6, 19.6)	< 0.001
Platelet (K/uL)	186.0 (149.0, 235.0)		199.0 (154.0, 257.0)	< 0.001
Glucose (mgl/L)	116.0 (103.0, 130.0)		171.0 (147.0, 209.0)	< 0.001
HbA1c (%)	5.8 (5.5, 6.4)		5.6 (5.3, 6.1)	< 0.001
Lactic acid(mmol/L)	1.8 (1.3, 2.6)		1.8 (1.3, 2.7)	0.39
Creatinine (mg/dL)	1.0 (0.8, 1.2)		1.1 (0.9, 1.6)	< 0.001
Potassium (mmol/L)	4.5 (4.2, 4.8)		4.5 (4.2, 5.0)	0.11
Sodium (mmol/L)	139.0 (137.0, 141.0)		140.0 (137.0, 142.0)	< 0.001
**Medicine and Outcomes**				
Anticoagulants, n (%)	2621 (91.7%)		1385 (94.4%)	< 0.001
VA, n (%)	129 (4.5%)		122 (8.3%)	< 0.001
VT, n (%)	105(3.7%)		91(6.2%)	< 0.001
VF, n (%)	38(1.3%)		47(3.2%)	< 0.001
Length of stay in ICU, days	2.2 (1.3, 4.1)		3.8 (2.0, 7.7)	< 0.001
ICU death, n (%)	95 (3.3%)		192 (13.1%)	< 0.001

Anticoagulants include heparin, warfarin, rivaroxaban and dabigatran

VA ventricular arrhythmia; CVICU Cardiac Vascular Intensive Care Unit; CCU Coronary Care Unit; MICU/SICU Medical/Surgical Intensive Care Unit; NSICU Neuro Surgical Intensive Care Unit; TSICU Trauma Surgical Intensive Care Unit; CAD coronary artery disease; COPD chronic obstructive pulmonary disease; DM diabetes mellitus; HbA1c glycosylated hemoglobin; SHR stress hyperglycemia ratio.

### Association between SHR and VA

Logistic regression analysis was performed to determine whether SHR provides additional diagnostic value beyond established hyperglycemia criteria for VA. The result of multivariate logistic regression analysis revealed that only SHR demonstrated statistical significance, indicating its superiority over prior definitions of higher average blood sugar in predicting VA among the ICU patients (**Supplemental Tables 2–3**).

**Fig. 2:**
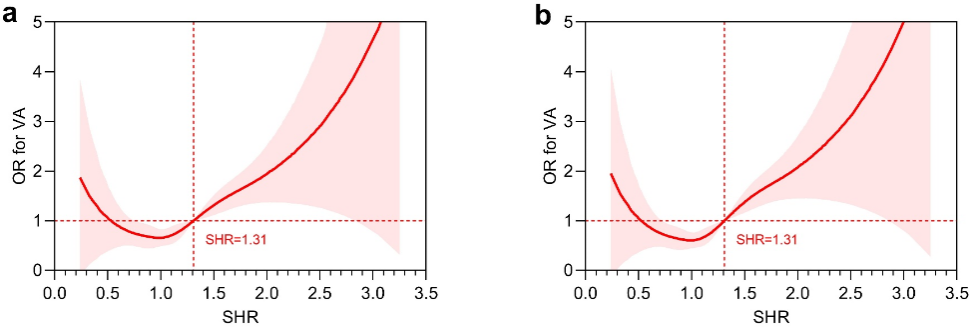
**Restricted cubic spline showing the dose-response relationship between stress hyperglycemia ratios and ventricular arrhythmia** (a) crude model and (b) adjusted model The dotted lines represent 95% confidence intervals, and the solid line represents odds ratios. The optimal cut-off value (1.31) of SHR determined by the receiver operating characteristic curve, was used as the reference. Correction variables include gender; age; body mass index; hypertension; diabetes mellitus; and coronary artery disease. OR odd ratio; SHR stress hyperglycemia ratio; VA ventricular arrhythmia

RCS was used to analyze the dose-response relationship between SHR on the first day of admission in the ICU and VA in critically ill patients. In the crude model, a “J-shaped” relationship was found between VA and SHR, where higher SHR levels (> 1.31) were associated with an increased risk of VA in the ICU (Fig. 2a). This relationship remained significant even after adjusting for traditional risk factors (Fig. 2b).

Results from the univariate logistic model analysis showed that the occurrence of VA was significantly associated with high SHR levels (OR 1.92, 95% CI: 1.48–2.47 *P* < 0.001). Furthermore, multivariate logistic model analysis, after adjusting for traditional risk factors such as BMI, gender, age, hypertension, DM, and CAD, demonstrated that high SHR levels were still a significant independent risk factor for VA [OR 2.07, 95% CI: 1.59–2.69 *P* < 0.001] (Table 2). Expanding upon this framework, this study also incorporated potential risk factors separately, including severity of illness score (SOFA SIRS OASIS), anticoagulants, insulin resistance (Triglyceride-Glucose Index), hyperglycemia, and inflammatory markers (Systemic immune-inflammation index) as adjusted variables. Despite the inclusion of these variables, our results consistently revealed a statistically significant correlation between high SHR levels and the incidence of VA (**Supplemental Tables 4–6**). When patients were stratified based on DM and a cut-off value of SHR, those with high SHR and without DM had a greater risk of VA [OR 2.22, 95% CI: 1.49–3.28] **(Table 2)** compared to the remaining groups. This relationship remained significant even after adjusting for variables [OR 2.38, 95% CI: 1.59–3.56]. However, this association was not statistically significant in the low SHR group.

In addition, we also examined the relationship between blood lipid markers and SHR and VA (**Supplemental Table 7**). Results revealed that total cholesterol (TC) levels were significantly associated with SHR and VA, with patients in the high-SHR group tending to have lower total cholesterol levels. Interestingly, in both high- and low-SHR groups, VA patients had lower TC levels than non-VA patients.

**Table 2:** Logistic regression model analysis showing the relationship of ventricular arrhythmia and SHR between the different groups

Group	Crude model		Adjust model			
	OR (95%CI)	*P* value	OR (95%CI)	*P* value		
patients stratified by SHR ^a^						
Low SHR (< 1.31)	Reference	-	Reference		-	
High SHR (≥ 1.31)	1.92 (1.48 to 2.47)	< 0.001	2.07 (1.59 to 2.69)		< 0.001	
patients stratified by SHR and DM ^b^						
Low SHR/DM	Reference	-	Reference		-	
Low SHR/non-DM	1.10(0.75 to 1.62)	0.640	1.10(0.74 to 1.63)		0.638	
High SHR/DM	1.69(1.05 to 2.73)	0.032	1.86(1.15 to 3.01)		0.012	
High SHR/ non-DM	2.22(1.49 to 3.28)	< 0.001	2.38(1.59 to 3.56)		< 0.001	

^a^: adjusted variables were the same as the restricted cubic spline analysis

^b^: adjusted variables were the same as the restricted cubic spline analysis but excluding diabetes mellitus SHR: stress hyperglycemia ratio; DM: diabetes mellitus; OR: Odd ratio; CI: confidence interval

### Subgroup analysis

**Fig. 3:**
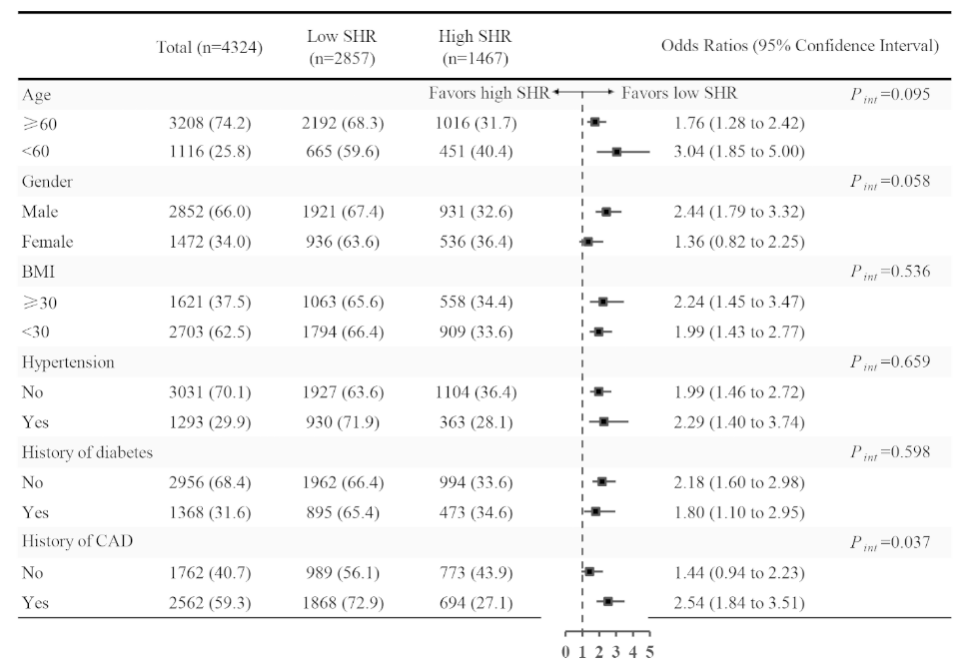
**Subgroup analysis to identify variables potentially impacting the association between stress hyperglycemia ratios and ventricular arrhythmia** BMI body mass index; CAD coronary artery disease; SHR stress hyperglycemia ratio; ROC receiver operating curve; VA ventricular arrhythmia

Subgroup analysis was performed to investigate the correlation between SHR and VA patients among stratified by various risk factors. A significant relationship was observed between the SHR and VA in male patients [OR 2.44, 95% CI: 1.79–3.32], those aged < 60 years [OR 3.04, 95% CI: 1.85–4.99], those aged ≥ 60years [OR 1.76, 95% CI: 1.28–2.41], those with a BMI ≥ 30 [OR 2.24, 95% CI: 1.44–3.46], and those with a BMI < 30 [OR 1.99, 95% CI: 1.43–2.71]. Furthermore, a significant association was found between SHR and VA in patients with hypertension [OR 2.28, 95% CI: 1.39–3.73] and those without hypertension [OR 1.99, 95% CI: 1.46–2.71], as well as patients with DM [OR 1.79, 95% CI: 1.09–2.95] and those without DM [OR 2.18, 95% CI: 1.60–2.97] Notably, SHR had a greater predictive value among patients with CAD [OR 2.54, 95% CI: 1.84–3.51] **(**Fig. 3**).**

## Discussion

This study enrolled 4,324 critically ill patients admitted to the ICU and found that SHR is an independent biomarker of VA, with an optimal cut-off value of 1.31 The correlation remained significant even after adjusting for various variables. To our knowledge, this is the first study to identify a meaningful association between SHR and the incidence of VA in ICU patients. Our findings suggest that SHR might be a reliable predictor of VA among such patients. Our study demonstrated a “J-shaped” relationship between SHR and VA, consistent with previous descriptions of the relationship between SHR and other outcomes [6, 11]. Mild increases in SH have been shown to boost cellular glucose utilization in response to stress, and thus the J-shaped association of SHR with other outcomes may be expected [12]. However, our RSC model analysis did not show statistical significance for the lower SHR range, which may be due to the small sample size in this group. Further studies are required studies to confirm these findings. High levels of blood glucose, or SH, are a common occurrence in critically ill patients admitted to the ICU. This is because critical illnesses such as trauma, sepsis, and burns activate the sympathetic autonomic nervous system, which stimulates in the production of hormones such as epinephrine and glucagon [13]. These hormones can indirectly or directly antagonize insulin, resulting in insulin resistance and elevated blood glucose levels [13]. Furthermore, hyperglycemia exacerbates these processes by releasing more inflammatory cytokines, leading to a vicious cycle [14].

While SH is essential for survival, levels that exceed 11.1 mmol/L have been reported to be associated with poor prognosis [15, 16]. Numerous studies have shown that SH is linked to adverse outcomes in various diseases [17–19]. However, these studies have relied on blood glucose concentrations at admission and did not account for differences in patients’ history of blood glucose levels. [20, 21]. In addition to acute stress, hyperglycemia in hospitalized patients may also result from long-term poor glycemic control. Therefore, a combination of SHR and HbA1c has been proposed as a new indicator of SH [6]. HbA1c has low biological variability and is not affected by acute stress responses [14]. It should be noted that the definition of SHR in our study could better optimize and calculate the blood glucose status of each critically ill patient under stress. There is limited research on the relationship between SH and VA. It is thought that the pathogenesis of VA induced by SH may be due to increased radical production and sympathetic activity during stress reduced nitric oxide availability, and subsequent ventricular instability [22]. Previous studies have focused on patients with heart disease and measured blood glucose levels only at admission, without accounting for differences in chronic blood glucose levels for example, a study conducted in Spain found that elevated blood glucose levels on admission were associated with a more than two-fold increased risk of VA compared with patients to normal or lower blood glucose levels [23]. Similarly, Hoang V. Tran et al. [24] recently found that patient’s with high blood glucose levels on admission for acute myocardial infarction had a greater risk of developing VT during hospitalization. However, these studies measured the patient’s blood glucose concentration at admission (fasting or random blood glucose) without accounting for differences in chronic blood glucose level. Moreover, only patients with acute myocardial infarction and other heart diseases were included. VA caused by acute hyperglycemia cannot be ignored in critical illness, especially in patients without heart disease. Dongen et al. [25] found that high HbA1c levels were associated with an increased risk of VF among patients with and without cardiovascular disease. Therefore, it is essential to investigate the association between SHR and VA in ICU patients, particularly for early prevention and treatment [26].

Our study investigated the relationship between SHR and new-onset VA in ICU patients stratified by DM and a cut-off value of SHR. Our study findings suggested that patients without a previous history of DM may be at a higher risk for new-onset VA, especially in the high SHR group. This relationship was further confirmed in subgroup analyses of DM. The discrepancy between our results and previous studies may be because ICU patients with a history of DM may have received antidiabetic therapies [24, 27]. Additionally, in the presence of DM, there may be a “preconditioning phenomenon” that increases antioxidant defenses, and protects tissues from oxidative stress response caused by acute hyperglycemia [28]. Our subgroup analysis showed a significant interaction between SHR and CAD (*P* interaction = 0.037). It has been demonstrated that SH could raise the concentration of free fatty acids through oxidative stress response and insulin resistance, which could directly impact the heart’s normal rhythm, especially in patients with pre-existing myocardial ischemia [29, 30]. Therefore, severe SH may promote hemodynamic disturbance and myocardial electrical instability in intensive care patients with CAD. Notably, our study also found that the relationship between SHR and VA was more significant in younger patients. Patients with a high SHR were generally younger than those with a low SHR, consistent with findings of similar studies in patients with myocardial infarction or other heart diseases [11]. Young patients in the ICU may have a more pronounced immunoreaction and sympathetic activity in response to acute stress, which could increase the risk of VA. However, further studies are required to confirm these hypotheses.

Furthermore, this study has established an association between TC levels and VA. Previous studies have demonstrated the link between TC and the risk of atrial or VA [31]. Cholesterol is a crucial component of cell membrane formation. In myocardial cells, the absence of cholesterol may undermine the stability of the myocardial cell membrane, modify the permeability of the potassium channel, and accelerate the emergence of cardiac electrical dysfunction [32]. Therefore, cholesterol may exert an influence on the incidence of arrhythmia by regulating the lipid content of the cardiac membrane. [33]. However, further experimental approaches are needed to clarify this potential relationship.

This study had a larger primary sample size than earlier studies and included a diverse population of intensive care patients with different diagnoses and disease states, which enhances the clinical applicability of our findings. A significant strength of this study was the use of a readily available metric, SHR, to measure SH and determine its relationship with VA in ICU patients. As glucose and glycated hemoglobin are commonly measured in critically ill patients, the SHR can be easily calculated. However, the study has some limitations, including being a single-center, retrospective study, limited to patients in the MIMIC IV database, which may have resulted in selection bias. Additionally, a large number of patients were excluded due to missing HbA1c values, which may have introduced sample selection bias. The study did not measure SHR dynamically, and the impact of SHR variability on VA during hospitalization is unclear. Lastly, studies have shown that higher SH is associated with an increased risk of developing DM in the future [34], but our database did not include information on new DM during follow-up. Therefore, its impact on patient outcomes could not be assessed.

## Conclusions

High SHR was a marked risk predictor for higher VA risk in critically ill patients admitted to the ICU. Critically ill patients could benefit from this simple indicator for predicting VA earlier. Therefore, our study underscores the potential clinical utility of SHR as a simple and accessible risk indicator for predicting VA in critically ill patients.

## Electronic supplementary material

Below is the link to the electronic supplementary material.

**Additional File 1:** Baseline characteristics of patients based on the ventricular arrhythmia.

## Data Availability

The data that support the findings of this study are available from https://mimic.mit.edu/. Although the database is publicly and freely available, researchers must complete the National Institutes of Health’s web-based course known as Protecting Human Research Participants to apply for permission to access the database. Data are available to researchers on request for purposes of reproducing the results or replicating the procedure by directly contacting the corresponding author.

## Abbreviations

BMI: body mass index
CVICU: Cardiac Vascular Intensive Care Unit
CCU: Coronary Care Unit
CAD: coronary artery disease
COPD: chronic obstructive pulmonary disease
CI: confidence interval
DM: diabetes mellitus
HbA1c: glycosylated hemoglobin
ICU: Intensive care unit
MICU/SICU: Medical/Surgical Intensive Care Unit
MIMIC: Medical information mart for intensive care
NSICU: Neuro Surgical Intensive Care Unit
OR: odd ratio
RCS: Restricted cubic spline
SH: stress hyperglycemia
SHR: stress hyperglycemia ratio
SPO2: blood oxygen saturation
TC: Total cholesterol
TSICU: Trauma Surgical Intensive Care Unit
VA: ventricular arrhythmia
VT: ventricular tachycardia
VF: ventricular fibrillation.
