# Correction: Association of hyperglycemia ratio and ventricular arrhythmia in critically ill patients admitted to the intensive care unit

**DOI:** 10.1186/s12872-023-03296-7

**Published:** 2023-05-19

**Authors:** Hechen Shen, Song Wang, Chong Zhang, Wenqing Gao, Xiaoqiong Cui, Qiang Zhang, Yuheng Lang, Meng Ning, Tong Li

**Affiliations:** 1 The Third Central, Clinical College of Tianjin Medical University, Tianjin, China; 2 Department of Heart Center, The Third Central Hospital of Tianjin, Tianjin, China; 3 Tianjin Key Laboratory of Extracorporeal Life Support for Critical Diseases, Tianjin, China; 4 Tianjin ECMO Treatment and Training Base, Tianjin, China; 5 Artificial Cell Engineering Technology Research Center, Tianjin, China; 6 School of Medicine, Nankai University, Tianjin, China; 7 Nankai University Affiliated Third Center Hospital, Nankai University, Tianjin, China

**Correction: BMC Cardiovasc Disord 23, 215 (2023)**

**https://doi.org/10.1186/s12872-023-03208-9**

Following publication of the original article [1], the below equal contribution statement has been included to the online article.

Hechen Shen, Song Wang and Chong Zhang have contributed equally to this work

The original article has been corrected.

## References

[1] Shen H (2023). Association of hyperglycemia ratio and ventricular arrhythmia in critically ill patients admitted to the intensive care unit. BMC Cardiovasc Disord.

