# Supplementary material for: Association of hyperglycemia ratio and ventricular arrhythmia in critically ill patients admitted to the intensive care unit
Source: BMC Cardiovasc Disord. 2023 Apr 28;23:215. doi: 10.1186/s12872-023-03208-9 (PMC10148444; doi:10.1186/s12872-023-03208-9)
Supplement: Supplementary file 1 — Additional File 1: Baseline characteristics of patients based on the ventricular arrhythmia. [file 12872_2023_3208_MOESM1_ESM.docx]

**Supplemental Table 1:** Baseline characteristics of patients based on the ventricular arrhythmia.

| Characteristics | Non-VA (n=4073) | VA (n=251) | *P* value |
| --- | --- | --- | --- |
| **Demographics** |  |  |  |
| Age (years) | 67.0±13.2 | 65.7±13.2 | 0.130 |
| Sex, male, n (%) | 2667 (65.5%) | 185 (73.7%) | 0.008 |
| Race, n (%) |  |  | 0.093 |
| White | 2611(64.1%) | 172(68.5%) |  |
| Hispanics | 18(0.4%) | 3(1.2%) |  |
| Black | 243(6.0%) | 9(3.6%) |  |
| Others | 1201(29.5%) | 67(26.7%) |  |
| Body mass index (kg/m2) | 29.2 (6.4) | 28.6 (5.8) | 0.160 |
| First Care Unit, n (%) |  |  | <0.001 |
| CVICU | 2592 (63.6%) | 130 (51.8%) |  |
| CCU | 417 (10.2%) | 90 (35.9%) |  |
| MICU/SICU | 602 (14.8%) | 23 (9.2%) |  |
| NSICU | 288 (7.1%) | 2 (0.8%) |  |
| TSICU | 174 (4.3%) | 6 (2.4%) |  |
| **Vital signs** |  |  |  |
| Respiratory Rate(beats/minute) | 18.1 (16.4, 20.3) | 18.2 (16.4, 20.4) | 0.69 |
| Heart rate (beats/minute) | 81.0 (74.0, 89.2) | 79.4 (71.5, 87.0) | 0.022 |
| Systolic blood pressure(mmHg) | 113.9 (106.8, 122.6) | 110.4 (103.8, 118.7) | 0.001 |
| Diastolic blood pressure(mmHg) | 59.3 (53.8, 65.9) | 60.0 (52.7, 66.8) | 0.85 |
| SpO2 (%) | 97.7 (96.4, 98.7) | 97.7 (96.6, 99.0) | 0.15 |
| **Comorbidities** |  |  |  |
| CAD n (%) | 2398 (58.9%) | 164 (65.3%) | 0.043 |
| COPD n (%) | 211 (5.2%) | 17 (6.8%) | 0.27 |
| DM n (%) | 1297 (31.8%) | 71 (28.3%) | 0.24 |
| Hypertension n (%) | 1220 (30.0%) | 73 (29.1%) | 0.77 |
| Congestive heart failure, n (%) | 1300 (31.9%) | 133 (53.0%) | <0.001 |
| Peripheral vascular disease, n (%) | 614 (15.1%) | 36 (14.3%) | 0.75 |
| **Scoring systems** |  |  |  |
| SOFA scores | 5(3,7) | 6(4,10) | <0.001 |
| OASIS scores | 32(26,38) | 34(27,41) | 0.002 |
| SIRS scores | 3(2,3) | 3(2,3) | 0.695 |
| SHR | 1.1 (0.9, 1.3) | 1.2 (1.0, 1.5) | <0.001 |
| **Laboratory tests** |  |  |  |
| Hemoglobin (g/dL) | 11.6 (10.3, 13.0) | 11.9 (10.3, 13.6) | 0.037 |
| White blood cell (K/µL) | 14.5 (10.9, 18.8) | 15.8 (11.5, 19.6) | 0.017 |
| Platelet (K/uL) | 189.0 (150.0, 242.0) | 206.5 (162.0, 261.0) | 0.002 |
| Glucose (mgl/L) | 128.0 (109.0, 156.0) | 140.0 (115.0, 187.0) | <0.001 |
| HbA1c (%) | 5.8 (5.5, 6.3) | 5.7 (5.5, 6.2) | 0.43 |
| Lactic acid (mmol/L) | 1.8 (1.3, 2.6) | 1.9 (1.3, 3.0) | 0.021 |
| Creatinine (mg/dL) | 1.0 (0.8, 1.3) | 1.1 (0.9, 1.5) | <0.001 |
| Potassium (mmol/L) | 4.5 (4.2, 4.9) | 4.7 (4.3, 5.0) | 0.002 |
| Sodium (mmol/L) | 139.0 (137.0, 141.0) | 140.0 (137.0, 142.0) | 0.191 |

*VA*: ventricular arrhythmia; *CVICU*: Cardiac Vascular Intensive Care Unit; *CCU*: Coronary Care Unit; *MICU/SICU*: Medical/Surgical Intensive Care Unit; *NSICU*: Neuro Surgical Intensive Care Unit; *TSICU*: Trauma Surgical Intensive Care Unit; *CAD*: coronary artery disease; *COPD*: chronic obstructive pulmonary disease; *DM*: diabetes mellitus; *HbA1c*: glycosylated hemoglobin; *SHR*: stress hyperglycemia ratio.

**Supplemental Table 2:** Univariate logistic regression analysis of SHR and the logarithmic transformation of blood glucose for VA.

| Variables | OR（95%CI） | *P* value |
| --- | --- | --- |
| Low SHR (<1.31) | Reference |  |
| High SHR (≥1.31) | 1.92 (1.48 to 2.47) | <0.001 |
| Glucose, (Log) | 1.41(1.02 to 1.95) | 0.037 |

**Supplemental Table 3:** Multivariate logistic regression analysis of SHR and the logarithmic transformation of blood glucose for VA.

| Variables | OR（95%CI） | *P* value |
| --- | --- | --- |
| High SHR (≥1.31) | 2.01(1.53 to 2.63) | <0.001 |
| Glucose, (Log) | 1.23(0.86 to 1.77) | 0.258 |
| Age | 0.99(0.98 to 1.00) | 0.108 |
| Gender | 1.36(1.01 to 1.84) | 0.042 |
| Body mass index | 0.98(0.96 to 1.00) | 0.130 |
| Hypertension | 1.03(0.77 to 1.37) | 0.884 |
| Coronary artery disease | 1.54(1.15 to 2.06) | 0.004 |
| Diabetes mellitus | 0.83(0.62 to 1.11) | 0.202 |

Adjusted variables including gender, age, body mass index, hypertension, diabetes mellitus and coronary artery disease. *SHR*: stress hyperglycemia ratio.

**Supplemental Table 4:** Logistic regression model of the relationship between ventricular arrhythmia and SHR after incorporating corrected variables for severity of illness score

| Group | Crude model | | Adjust model ^a^ | |
| --- | --- | --- | --- | --- |
|  | OR（95%CI） | *P* value | OR（95%CI） | *P* value |
| Low SHR (<1.31) | Reference |  | Reference |  |
| High SHR (≥1.31) | 1.92 (1.48 to 2.47) | <0.001 | 1.86（1.42-2.44） | <0.001 |

^a^ Correction variables include gender; age; body mass index; hypertension; diabetes mellitus coronary artery disease; **SOFA; SIRS and OASIS**. *SHR*: stress hyperglycemia ratio.

**Supplemental Table 5:** Logistic regression model of the relationship between ventricular arrhythmia and SHR after incorporating corrected variables for anticoagulants

| Group | Crude model | | Adjust model ^a^ | |
| --- | --- | --- | --- | --- |
|  | OR（95%CI） | *P* value | OR（95%CI） | *P* value |
| Low SHR (<1.31) | Reference |  | Reference |  |
| High SHR (≥1.31) | 1.92 (1.48 to 2.47) | <0.001 | 2.03 (1.56-2.64) | <0.001 |

^a^ Correction variables include gender; age; body mass index; hypertension; diabetes mellitus; coronary artery disease and **anticoagulants (heparin, warfarin, rivaroxaban and dabigatran)**. *SHR*: stress hyperglycemia ratio.

**Supplemental Table 6:** Logistic regression model of the relationship between ventricular arrhythmia and SHR after incorporating corrected variables for insulin resistance, inflammation and hyperglycemia.

| Variables | OR（95%CI） | *P* value |
| --- | --- | --- |
| Low SHR (<1.31) | Reference |  |
| High SHR (≥1.31) | 2.25 (1.36-3.73) | 0.002 |
| Age | 0.98(0.96-0.99) | 0.005 |
| Gender | 2.11(1.19-3.74) | 0.011 |
| Body mass index | 0.99(0.96-1.03) | 0.610 |
| Hypertension | 1.00(0.59-1.71) | 0.989 |
| Coronary artery disease | 2.39(1.45-3.91) | <0.001 |
| Diabetes mellitus | 0.79(0.46-1.37) | 0.406 |
| TyG index | 0.92(0.63-1.33) | 0.661 |
| SII index | 1.00(0.99-1.00) | 0.410 |
| Glucose, (Log) | 2.00(0.61-6.64) | 0.256 |

Adjusted variables including gender, age, body mass index, hypertension, diabetes mellitus, coronary artery disease, **insulin resistance (TyG index), inflammation (SII index) and hyperglycemia.** TyG index values were derived from 2114 participants due to the partially missing data for some patients; SII index values were derived from 2834 participants due to the partially missing data for some patients. *SHR*: stress hyperglycemia ratio; *TyG*: Triglyceride-Glucose index; *SII*: Systemic immune-inflammation index.

**Supplemental Table 7:** Association of lipid profile with both SH and VA

| Lipid level | Low SHR/non-VA | High SHR/non-VA | Low SHR/VA | High SHR/VA | *P* valve |
| --- | --- | --- | --- | --- | --- |
| N | 2728 | 1345 | 129 | 122 |  |
| Triglyceride (mg/dl) ^a^ | 112.0 (82.0, 167.0) | 115.0 (81.0, 176.0) | 108.0 (77.0, 145.0) | 110.5 (75.0, 145.0) | 0.19 |
| Total cholesterol (mg/dl) ^b^ | 158.0 (128.0, 192.0) | 152.0 (121.0, 189.0) | 150.0 (123.0, 184.0) | 143.0 (117.5, 171.0) | 0.011 |
| High-density lipoprotein (mg/dl) ^c^ | 45.0 (36.0, 56.0) | 45.0 (35.0, 56.0) | 44.5 (36.0, 53.5) | 42.0 (32.0, 54.0) | 0.42 |
| Low density lipoprotein (mg/dl) ^d^ | 96.0 (76.0, 112.0) | 86.0 (68.0, 116.5) | 94.0(74.0, 106.0) | 105.0 (68.0, 145.0) | 0.21 |

**^a^** Triglyceride values were derived from 2114 participants due to the partially missing data for some patients; **^b^** Total cholesterol values were derived from 2043 participants due to the partially missing data for some patients; **^c^** High-density lipoprotein values were derived from 1990 participants due to the partially missing data for some patients; **^d^** Low density lipoprotein values were derived from 424 participants due to the partially missing data for some patients. *VA* ventricular arrhythmia, *SHR*: stress hyperglycemia ratio.
